# Risk of uterine leiomyomata with menstrual and reproductive factors in premenopausal women: Korea nurses’ health study

**DOI:** 10.1186/s12905-023-02447-4

**Published:** 2023-06-09

**Authors:** Sihan Song, Soojin Park, Bo Mi Song, Jung Eun Lee, Chiyoung Cha, Hyun-Young Park

**Affiliations:** 1 Division of Population Health Research, Department of Precision Medicine, Korea National Institute of Health, Cheongju, 28159 Republic of Korea; 2 Department of Food and Nutrition, Seoul National University, Seoul, 08826 Republic of Korea; 3 College of Nursing, System Health & Engineering major in Graduate School, Ewha Womans University, Seoul, 03760 Republic of Korea; 4 Department of Precision Medicine, Korea National Institute of Health, Cheongju, 28159 Republic of Korea

**Keywords:** Uterine leiomyomata, Menarche, Menstrual cycles, Reproductive health, Prospective studies

## Abstract

**Background:**

Uterine leiomyomata (UL) are benign smooth muscle tumors that may cause significant morbidity in women of reproductive age. This study aimed to investigate the relationship of menstrual and reproductive factors with the risk of UL in premenopausal women.

**Methods:**

This prospective study included 7,360 premenopausal women aged 22–48 years who were part of the Korea Nurses’ Health Study. Information on the menstrual cycle and reproductive history was assessed between 2014 and 2016, and self-reported cases of UL were obtained through 2021. Cox proportional hazards models were used to estimate the hazard ratios (HRs) and 95% confidence intervals (CIs).

**Results:**

During 32,072 person-years of follow-up, 447 incident cases of UL were reported. After adjusting for other risk factors, women with late age at menarche had a lower incidence of UL (≥ 16 vs. 12–13 years: HR 0.68; 95% CI 0.47–0.99; p for trend = 0.026). The risk of UL was inversely associated with current menstrual cycle length (≥ 40 or too irregular to estimate vs. 26–31 days: HR 0.40; 95% CI 0.24–0.66) and cycle length at ages 18–22 years (HR 0.45; 95% CI 0.31–0.67; p for trend < 0.001, each). Parous women had lower risk of UL than nulliparous women (HR 0.40; 95% CI 0.30–0.53) and women who were aged 29–30 years at first birth had a lower risk of UL than those who were aged ≤ 28 years at first birth (HR 0.58; 95% CI 0.34–0.98). There was no significant association of the number of births or breastfeeding with the risk of UL among parous women. Neither a history of infertility nor oral contraceptive use was associated with the risk of UL.

**Conclusions:**

Our results suggest that age at menarche, menstrual cycle length, parity, and age at first birth are inversely associated with the risk of UL in premenopausal Korean women. Future studies are warranted to confirm the long-term effects of menstrual and reproductive factors on women’s health.

## Background

Uterine leiomyomata (UL), also known as myomas or fibroids, are benign tumors arising from the smooth muscle cells of the uterus [1]. Clinical symptoms include menorrhagia, pelvic pain, infertility, and pregnancy complications; symptomatic UL are the most common indication for hysterectomy [1, 2]. Globally, the age-standardized incidence rate of UL has steadily increased from 225.67 to 241.18 per 100,000 women between 1990 and 2019, respectively [3]. The occurrence of UL in the general population is likely to be underestimated because it is often asymptomatic and diagnosed incidentally during examination or surgery [1]. In an ultrasound screening study, the rates of self-reported and newly diagnosed UL in premenopausal women in the US were 35% and 51%, respectively [4]. The incidence of UL increases with age until menopause, and African-Americans have a higher risk of UL than other ethnicities [4–6]. Although its etiology remains largely unknown, UL are thought to have a genetic basis and are influenced by hormones and growth factors [1, 7, 8]. Reproductive factors, including early menarche and nulliparity, are recognized UL risk factors, and lifestyle factors such as obesity and alcohol drinking are possible risk factors for UL [9–11]. According to a recent report from the National Health Insurance Service–National Sample Cohort (NHIS-NSC), the cumulative incidence from 2003 to 2013 was 12.2% in Korean women, and the increase in the annual incidence was higher in younger ages [12]. The prevalence of UL in Korean women was reported as 9.0% in a self-administered online survey of reproductive age women conducted in 2009 [13] and 37.5% in a pelvic ultrasound study of middle-aged women conducted between 2005 and 2008 [14]. Decreased age at menarche [15], birth rate [16], and increased health examination rate [17] may explain the increasing prevalence of UL in Korea. However, more prospective data are required to provide evidence for UL prevention and treatment.

Current evidence on the risk factors for UL is largely derived from African-American and White women [10, 11]. Given the inconsistent results of previous cross-sectional [13, 18] and case-control [19–22] studies in Asian populations, prospective data are needed to investigate the potential role of reproductive factors in the UL etiology.

Therefore, in this prospective cohort study of female nurses in Korea, we aimed to provide evidence of the association between menstrual and reproductive factors and risk of UL in premenopausal women.

## Methods

### Study population

The Korea Nurses’ Health Study (KNHS) is an ongoing prospective study of female Korean nurses [23]. A total of 20,613 women aged 20–45 years completed their first online survey (Module 1) between July 2013 and November 2014. Information on demographics, lifestyle, reproductive factors, and disease history were collected in Module 1. Six online surveys (Modules 2–7) were subsequently opened to participants between March 2014 and September 2019, and participants continued to be followed up via annual questionnaires from Module 8, which started in October 2019. Several questions such as disease history and job status were repeated in the modules. A detailed description of this cohort has been published previously [23]. Information on menstrual characteristics, oral contraceptive use, and a recent gynecological examination was first collected in Module 3, which was opened in November 2014. In this study, we included women who completed Module 3 before the opening Module 5 (November 2016), to ensure sufficient follow-up time by Module 9 (April 2021). Furthermore, this study participants were restricted to premenopausal women because UL develop during the reproductive years and commonly regress after menopause [7].

**Fig. 1:**
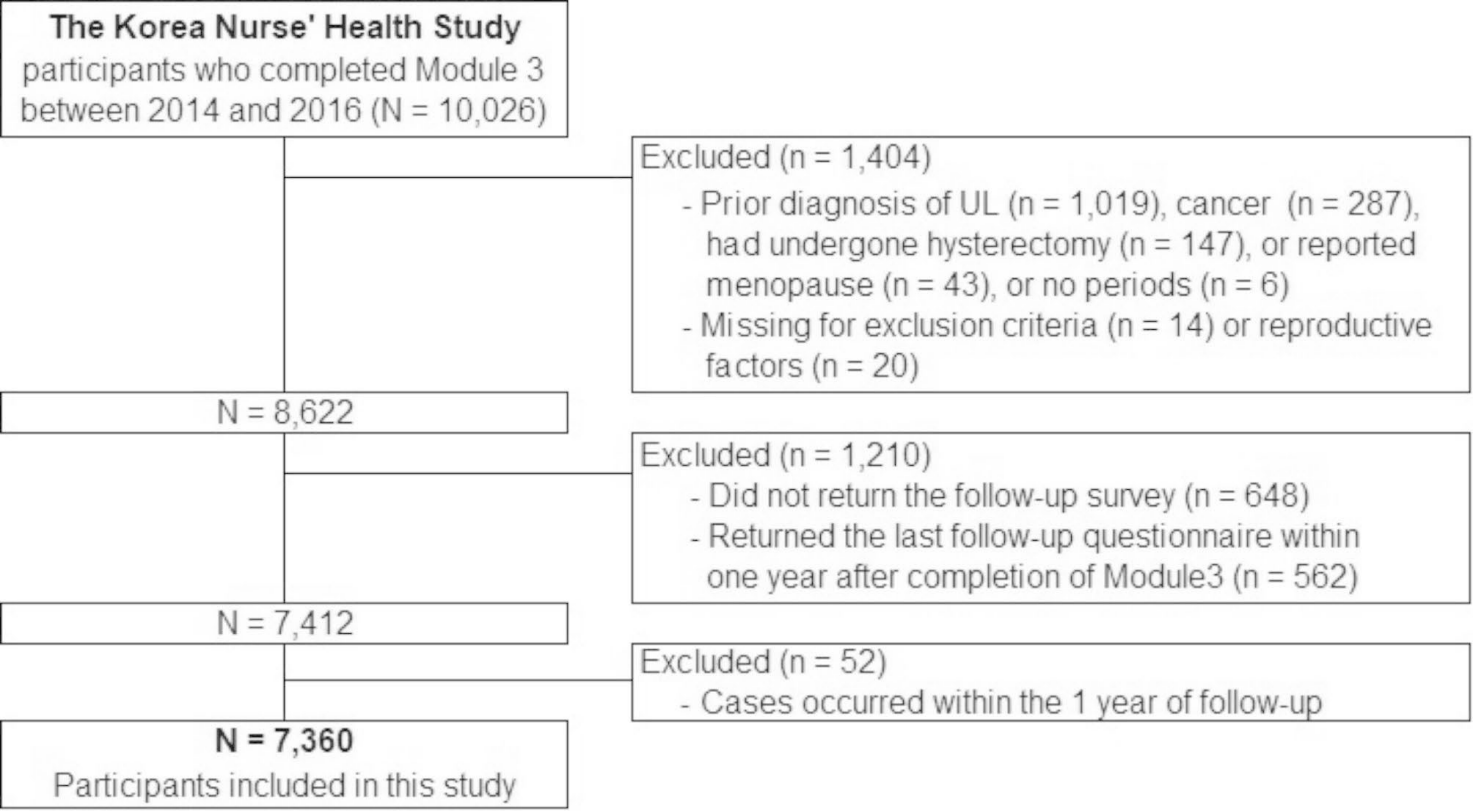
Flowchart of the study participants

The flowchart of participant selection in this study is shown in Fig. 1. Of the 10,026 women who completed Module 3 between 2014 and 2016, we excluded those who were diagnosed with UL (n = 1,019) or cancer (n = 287); had undergone hysterectomy (n = 147); or reported menopause (n = 43) or no periods (n = 6). Women with missing data on exclusion criteria (n = 14) or reproductive factors (n = 20) were also excluded. Furthermore, women who did not return the follow-up survey (n = 648) or those who returned the last follow-up survey within one year after completion of Module 3 (n = 562) were excluded, leaving 7,412 women followed up from 2014 to 2021. Similar reproductive characteristics were observed between the included women and dropouts (data not shown). We further excluded those with cases of UL (n = 52) that occurred within one year of follow-up to reduce the possibility of reverse causation. Finally, 7,360 premenopausal women aged 22–48 years at baseline were included in this study.

### Exposure assessment and covariates

Participants were asked about their age at menarche, time to regular menstrual cycles, and menstrual cycle patterns. In this study, we categorized age at menarche as ≤ 11, 12–13, 14–15, and ≥ 16 years and time to regular menstrual cycles as ≤ 1, 2–4, and ≥ 5 years or always irregular. Questions on menstrual patterns included cycle regularity and length at baseline and in early adulthood (ages 18–22 years). We categorized menstrual cycle regularity as very regular, regular, usually regular, and always regular and cycle length as < 26, 26–31, 32–39, and ≥ 40 days or too irregular to estimate. Data on parity history (defined as the total number of live births: nulliparous, 1, 2, and ≥ 3), age at first birth, and total months of breastfeeding were collected in Module 1 and during follow-up modules. Participants were asked if they had tried to conceive for at least one year (history of infertility) and, if so, whether they had consulted a physician to seek help. Information on the use of oral contraceptives for at least two months and Pap smear screening in the past two years was also obtained.

Data on anthropometric measurements and lifestyle factors, including smoking status and alcohol drinking, were collected during the initial baseline survey. The body mass index (BMI) was calculated as body weight divided by height squared (kg/m^2^). BMI was categorized into four groups (< 18.5, 18.5–22.9, 23.0–24.9, ≥ 25 kg/m^2^) according to the World Health Organization Asia-Pacific guidelines [24]. Perceived stress was assessed using the 4-item perceived stress scale (score range 0–16, higher scores indicate greater stress) [25]. Data on occupational factors, including frequency of rotating night shifts and hours of standing work, were also collected. Data on blood pressure and antihypertensive medication use were collected in Module 3. Participants who reported the use of antihypertensive medications were asked to report their most recent blood pressure without medication. Systolic blood pressure (SBP) was categorized as < 105, 105–114, 115–124, 125–134, and ≥ 135 mmHg.

### Outcome assessment and follow-up

In the follow-up surveys, participants were asked if they had been diagnosed with UL by a physician (Modules 4, 5, 7–9) and had undergone myomectomy (Modules 4, 5, 8, 9). Thus, the participants were asked to report their calendar year of diagnosis and/or surgery. Updated information on cancer diagnosis, hysterectomy, and menopausal status was also obtained from the follow-up questionnaires. The person-years were calculated from the date of return of Module 3 to the date of diagnosis of UL, cancer, hysterectomy, menopause, or last returned Module, whichever came first. The index date was defined as the midpoint of the calendar year. If the year of diagnosis, surgery, or menopause was the same as the survey year, the index date was defined as the midpoint of the survey date. 85% of the participants returned Module 8 or 9 during a median follow-up of 4.6 years.

### Statistical analysis

Descriptive statistics are presented as median and interquartile range (IQR) or percentages. Cox proportional hazards models were used to estimate age- and multivariable-adjusted hazard ratios (HRs) and 95% confidence intervals (CIs) of the associations between the reproductive factors of interest and UL risk. Multivariable models adjusted for age in years, age at menarche, menstrual cycle length, parity history, BMI, SBP, and a recent gynecological examination (Pap smear screening). Smoking, alcohol drinking, perceived stress, and occupational factors were not included in the final model, because these variables did not markedly change the estimates. P-values for the trend were calculated by assigning the median value (or mid-point) of each category of exposures in the model as a continuous variable. Associations of the number of births, age at first birth, and total duration of breastfeeding with UL risk were examined among parous women. The proportional hazard assumption was tested using time-dependent interaction terms, and no violation of this assumption was found. To explore the possibility of detection bias, all analyses were repeated in a subgroup of women who reported a recent gynecological examination (n = 2,924). Sensitivity analyses were performed by excluding women with a cycle length > 50 days or too irregular to estimate from the cycle length analyses and excluding those with a history of infertility from the parity analysis.

Statistical significance was defined as a two-sided p-value of < 0.05. All the analyses were performed using SAS version 9.4 (SAS Institute Inc., Cary, NC, USA).

## Results

During the 32,072 person-years of follow up, 447 new cases of UL were reported with a cumulative incidence of 6.1%. Approximately 20% of incident cases underwent myomectomy or hysterectomy. For the 7,360 participants included in this analysis, the median age at baseline was 29 years (IQR, 26–35 years) (Table 1). About half of the women had menarche before age 14 years, 51% had ≤ 1 years to regular menstrual cycles, 76% had very regular or regular cycles, and 59% had menstrual cycle lengths of 26–31 days. The menstrual patterns in early adulthood were similar to the current patterns (data not shown). Overall, 31% of the women were parous (median age at first birth, 30 years); of these, 50% reported having breastfed for at least 6 months. 6% of the women reported that they had tried to conceive for at least one year, 7% reported oral contraceptive use for at least two months, and 40% had a Pap smear within the past two years.

**Table 1:** Baseline characteristics of study participants

Characteristics	N = 7,360
Age (median years, IQR)	29 (26–35)
Age at menarche, years (%)	
≤ 11	4.9
12–13	44.8
14–15	40.8
≥ 16	9.5
Time to regularity, years (%)	
≤ 1	51.0
2–4	21.2
≥ 5 or always irregular	27.8
Menstrual cycle regularity (%)	
Very regular	31.6
Regular	44.3
Usually irregular	17.6
Always irregular	6.5
Menstrual cycle length, days (%)	
< 26	9.1
26–31	59.2
32–39	23.2
≥ 40 or too irregular to estimate	8.5
Parous (%)	31.5
Age at first birth (median years, IQR)	30 (28–32)
Duration of breastfeeding, ≥ 6 months (%)	50.1
Attempting to conceive, ≥ 1 year (%)	5.8
Oral contraceptives use, ≥ 2 months (%)	6.7
Age at first use (median years, IQR)	26 (24–29)
Pap spear screening within past 2 years (%)	39.7
Ever smokers (%)	2.6
Alcohol consumption, ≥ 1 drink/day (%)	7.6
Body mass index, kg/m^2^ (%)	
< 18.5	15.6
18.5–22.9	66.7
23–24.9	10.0
≥ 25	7.7
Perceived stress (scores, IQR)	7 (5–8)
Antihypertensive medication use (%)	0.5
Systolic blood pressure, mmHg (%)	
< 105	21.5
105–114	41.4
115–124	27.6
125–134	7.9
≥ 135	1.6
Rotating night shift, nights/month (%)	
None	31.1
< 7	36.8
≥ 7	32.1
On feet at work, hours/day (%)	
≤ 4	33.4
5–8	45.2
≥ 9	21.3

IQR, interquartile range (25th–75th percentile)

The risk of UL was inversely associated with age at menarche (Table 2). Compared with women aged 12–13 years at menarche, those aged 14–15 years (multivariable-adjusted HR 0.80; 95% CI 0.65–0.98) and ≥ 16 years at menarche (HR 0.68; 95% CI 0.47–0.98) had lower risks of UL (p for trend = 0.026). Time to regular cycles and regularity of current cycles were not associated with UL risk. However, irregular cycles in early adulthood were associated with a reduced risk of UL (always irregular vs. regular: HR, 0.61; 95% CI 0.39–0.97). Current and early adulthood cycle lengths were inversely associated with UL risk (p for trend < 0.001, respectively). Compared with women with a cycle length of 26–31 days, those with a cycle length of > 40 days or those with too irregular to estimate cycle lengths had a lower UL risk (HR 0.40; 95% CI 0.24–0.66). At ages 18–22 years, those with a cycle length of 32–39 days (HR 0.73; 95% CI 0.56–0.94) and ≥ 40 days or too irregular to estimate (HR 0.45; 95% CI 0.31–0.67) had lower risk of UL than those with a length of 26–31 days. Associations were robust when we restricted analyses to those who with a cycle length ≤ 50 days. The HRs (95% CIs) comparing cycle lengths of 40–50 with 26–31 days were 0.55 (0.30–1.00) for current and 0.42 (0.22–0.82) for early adulthood.

**Table 2:** Association between menstrual characteristics and risk of uterine leiomyomata

Characteristics (N = 7,360)	Person-years	Cases	Age-adjusted HR (95% CI)	Multivariate HR (96% CI)
Menarche age, years ^a^				
≤ 11	1547	22	0.96 (0.62–1.49)	0.96 (0.62–1.48)
12–13	14,341	227	Reference	Reference
14–15	13,178	165	0.75 (0.61–0.92)	0.80 (0.65–0.98)
≥ 16	3006	33	0.65 (0.45–0.94)	0.68 (0.47–0.99)
P for trend			0.007	0.026
Time to regular cycles, years^b^				
≤ 1	16,326	242	Reference	Reference
2–4	6829	94	0.96 (0.75–1.21)	0.92 (0.72–1.17)
≥ 5 or always irregular	8916	111	0.89 (0.71–1.12)	0.87 (0.70–1.10)
P for trend			0.341	0.253
Menstrual cycle regularity^b^				
Cycle regularity				
Very regular	9748	157	1.03 (0.84–1.27)	1.03 (0.84–1.27)
Regular	13,669	202	Reference	Reference
Usually irregular	5420	59	0.78 (0.58–1.04)	0.77 (0.57–1.03)
Always irregular	1998	22	0.79 (0.51–1.23)	0.78 (0.50–1.21)
P for trend			0.064	0.052
Cycle regularity ages 18–22 years				
Very regular	8423	142	1.11 (0.90–1.37)	1.09 (0.88–1.35)
Regular	14,890	214	Reference	Reference
Usually irregular	6277	69	0.78 (0.60–1.03)	0.78 (0.59–1.02)
Always irregular	2310	20	0.62 (0.39–0.97)	0.61 (0.39–0.97)
P for trend			0.002	0.003
Menstrual cycle length^b^				
Cycle length, days				
< 26	2793	42	0.95 (0.69–1.32)	0.96 (0.69–1.32)
26–31	18,224	291	Reference	Reference
32–39	7154	91	0.85 (0.67–1.08)	0.85 (0.67–1.08)
≥ 40 or too irregular to estimate	2663	16	0.41 (0.25–0.68)	0.40 (0.24–0.66)
P for trend			0.001	< 0.001
Cycle length ages 18–22 years, days				
< 26	2700	38	0.92 (0.66–1.29)	0.91 (0.65–1.28)
26–31	19,205	310	Reference	Reference
32–39	6225	70	0.71 (0.55–0.92)	0.73 (0.56–0.94)
≥ 40 or too irregular to estimate	3775	27	0.45 (0.31–0.67)	0.45 (0.31–0.67)
P for trend			< 0.001	< 0.001

^a^Adjusted for age at baseline, menstrual cycle length, parity history, body mass index, systolic blood pressure, and a recent gynecologic examination

^b^Adjusted for age at baseline, age at menarche, parity history, body mass index, systolic blood pressure, and a recent gynecologic examination

CI, confidence interval; HR, hazard ratio

Parous women had significantly lower risk of UL compared with nulliparous women (HR 0.40; 95% CI 0.30–0.53) (Table 3). This inverse association remained significant even after excluding women with a history of infertility (parous vs. nulliparous: HR, 0.38; 95% CI 0.29–0.51). Compared with women aged ≤ 28 years at first birth, those who were aged 29–30 at first birth had lower UL risk (HR 0.58; 95% CI 0.34–0.98). Among parous women, the number of births and total breastfeeding duration were not significantly associated with UL risk. No association was observed between either a history of infertility or oral contraceptive use and the risk of UL.

**Table 3:** Associations of reproductive history and oral contraceptive use with the risk of uterine leiomyomata

Characteristics (N = 7,360)	Person-years	Cases	Age-adjusted HR (95% CI)	Multivariate HR (96% CI)
Parity^a^				
Nulliparous	22,047	327	Reference	Reference
Parous	10,025	120	0.44 (0.34–0.56)	0.40 (0.30–0.53)
No. of births				
1	4214	37	Reference	Reference
2	5184	74	1.10 (0.72–1.68)	1.10 (0.72–1.68)
≥ 3	626	9	1.06 (0.50–2.23)	1.17 (0.55–2.48)
P for trend			0.737	0.625
Age at first birth, years				
≤ 28	3178	51	Reference	Reference
29–30	2231	19	0.57 (0.33–0.96)	0.58 (0.34–0.98)
≥ 31	3217	37	0.69 (0.45–1.06)	0.67 (0.44–1.03)
P for trend			0.113	0.086
Total breastfeeding, months				
0	837	17	Reference	Reference
1–5	3058	38	0.71 (0.40–1.26)	0.64 (0.36–1.14)
6–11	1849	25	0.83 (0.45–1.56)	0.80 (0.42–1.50)
≥ 12	2169	23	0.69 (0.36–1.32)	0.63 (0.33–1.21)
P for trend			0.472	0.415
Attempting to conceive (≥ 1 year)^b^				
No	30,186	428	Reference	Reference
Yes	1886	19	0.62 (0.39–0.98)	0.70 (0.44–1.12)
Consulting a physician				
No	957	9	Reference	Reference
Yes	929	10	1.05 (0.42–2.59)	1.36 (0.52–3.54)
Oral contraceptive use (≥ 2 months)^b^				
No	29,874	422	Reference	Reference
Yes	2195	25	0.88 (0.59–1.33)	0.98 (0.65–1.48)
Age at first use, years				
≤ 26	1323	15	Reference	Reference
> 26	752	9	0.96 (0.34–2.72)	0.75 (0.25–2.25)

^a^Adjusted for age at baseline, age at menarche, menstrual cycle length, body mass index, systolic blood pressure, and a recent gynecologic examination

^b^Adjusted for age at baseline, age at menarche, menstrual cycle length, parity history, body mass index, systolic blood pressure, and a recent gynecologic examination

CI, confidence interval; HR, hazard ratio

When we restricted the analyses to those who had a recent gynecological examination, the inverse associations of age at menarche, cycle length in early adulthood, and parity with UL risk remained significant (Table 4).

**Table 4:** Association between selected menstrual and reproductive factors and uterine leiomyomata risk among women reporting recent gynecologic examination

Characteristics (N = 2,924)	Person-years	Cases	Age-adjusted HR (95% CI)	Multivariate HR (96% CI)
Menarche age, years^a^				
≤ 11	568	11	1.16 (0.62–2.17)	1.19 (0.64–2.23)
12–13	5445	99	Reference	Reference
14–15	5498	69	0.65 (0.48–0.89)	0.70 (0.51–0.96)
≥ 16	1179	9	0.39 (0.20–0.77)	0.40 (0.20–0.79)
P for trend			< 0.001	0.001
Menstrual cycle length^b^				
Cycle length, days				
< 26	910	19	1.25 (0.77–2.02)	1.26 (0.77–2.05)
26–31	7271	120	Reference	Reference
32–39	2672	39	0.94 (0.65–1.35)	0.91 (0.63–1.31)
≥ 40 or too irregular to estimate	948	7	0.49 (0.23–1.05)	0.48 (0.22–1.03)
P for trend			0.04	0.03
Cycle length ages 18–22 years, days				
< 26	788	12	0.91 (0.51–1.64)	0.92 (0.51–1.67)
26–31	7933	138	Reference	Reference
32–39	2420	25	0.61 (0.40–0.93)	0.62 (0.40–0.95)
≥ 40 or too irregular to estimate	1472	12	0.48 (0.27–0.87)	0.49 (0.27–0.89)
P for trend			0.007	0.008
Parity^c^				
Nulliparous	5365	87	Reference	Reference
Parous	7326	101	0.51 (0.35–0.73)	0.52 (0.36–0.76)
Age at first birth, years				
≤ 28	2253	41	Reference	Reference
29–30	1607	16	0.61 (0.34–1.08)	0.62 (0.34–1.11)
≥ 31	2353	32	0.75 (0.47–1.19)	0.70 (0.44–1.12)
P for trend			0.263	0.157
Attempting to conceive (≥ 1 year)^d^				
No	11,213	174	Reference	Reference
Yes	1479	14	0.59 (0.34–1.01)	0.62 (0.36–1.08)
Oral contraceptive use (≥ 2 months)^d^				
No	11,631	178	Reference	Reference
Yes	1057	10	0.69 (0.37–1.32)	0.76 (0.40–1.45)

^a^Adjusted for age at baseline, menstrual cycle length, parity history, body mass index, and systolic blood pressure

^b^Adjusted for age at baseline, age at menarche, parity history, body mass index, and systolic blood pressure

^c^Adjusted for age at baseline, age at menarche, menstrual cycle length, body mass index, and systolic blood pressure

^d^Adjusted for age at baseline, age at menarche, menstrual cycle length, parity history, body mass index, and systolic blood pressure

CI, confidence interval; HR, hazard ratio

## Discussion

In this prospective study, we examined the association between reproductive factors and UL risk in premenopausal Korean women. We observed that the risk of UL was inversely associated with the age at menarche, menstrual cycle length, parity, and age at first birth. No associations were observed with either the number of birth, breastfeeding, history of infertility, or oral contraceptive use.

In this study, a later age at menarche was associated with a reduced risk of UL. Some cross-sectional [13, 18] or case-control studies [21, 22, 26] found no significant association between age at menarche and UL. However, two Korean case-control studies [21, 22] and one Japanese cross-sectional study [18] did not restrict the participants to women of reproductive age. Consistent with our study, epidemiologic studies conducted in the US suggest an inverse association between age at menarche and UL risk among premenopausal women [27–32]. In the Nurses’ Health Study (NHS) II that included a large cohort of female nurses who were predominantly Caucasian, a significant inverse association between age at menarche and UL risk was observed. The relative risks of UL for women aged ≥ 16 versus 12 years at menarche were 0.68 and 0.77 in the 4-year and 14-year follow-up, respectively [27, 29]. Similarly, the Black Women’s Health Study (BWHS) observed a 30% lower risk of UL among African-American women aged ≥ 15 versus < 11 years at menarche during a 4-year follow-up [28]. Moreover, in the “Right From the Start” study, ultrasound examinations were performed during early pregnancy to systematically screen for UL, and an association was observed between early age at menarche and the presence and number of UL [32]. Although the biologic mechanisms are not fully understood, women at an early age of menarche may have increased menstrual cycling and lifetime exposure to estrogens, which are thought to promote the growth of UL [27, 28]. Furthermore, early life factors that cause early menarche may be linked to the development of UL in adulthood [33].

Mitotic activity in the myometrium is higher during the luteal phase of the menstrual cycle [34]; therefore, frequent menstrual cycles may contribute to myoma formation. In this study, women with a long cycle length at baseline and those with long and/or irregular cycles at ages 18–22 years were less likely to develop UL. Our findings are biologically plausible, given that menstrual cycle length decreases with age until menopause, as the length of the follicular phase shortens [35]. Although the relationship between menstrual patterns and UL risk is less clear, studies on female nurses in the US and Japan have shown results consistent with those of our study. Long and/or irregular cycles were significantly associated with a lower risk of UL in the NHS II [29], and long cycle length at ages 18–22 was inversely associated with the odds of UL in the Japan Nurses’ Health Study [18]. In an online survey across eight countries, women with UL were more likely to report frequent periods than those without UL [13]. However, no such association was observed in a US case-control study [30] and an Italian cross-sectional study [36]. Further investigations are needed to clarify the role of menstrual patterns in UL development.

The inverse association between parity and UL risk in this study is consistent with the results of previous studies [19, 22, 27–29, 37–39]. Compared with nulliparous women, the risk reduction of UL in parous women ranges from 30 to 60%, and several studies have shown a decreased UL risk with an increasing number of births [27, 29, 37, 38]. The risk of UL among parous women did not decrease with each additional birth in our study, and a similar finding was observed in the BWHS [28]. Two US studies reported a lower incidence of UL with a later age at first birth and shorter time since last birth [28, 29]. Although the age range at first birth in our study was narrow, we observed a similar relationship with UL risk. There are several hypotheses regarding the role of pregnancy and childbirth in the occurrence of UL, including hormonal changes during pregnancy [34, 40, 41] and after birth [42], decreased lifetime exposure to ovarian hormones [43], and postpartum uterine involution and remodeling [44–46]. In NHS II, a risk reduction of UL was observed in women who breastfed for longer than 3 years, and this association may be partially explained by the postpartum amenorrhea [29]. However, consistent with our findings, no apparent association was observed between breastfeeding duration and UL risk in several studies [19, 26, 28].

In the present study, a history of infertility was not associated with the risk of UL. The presence of UL may be a cause rather than a consequence of infertility [33, 38]. Consistent with previous studies [27, 28], the inverse association with parity remained significant after excluding women with a history of infertility; therefore, reverse causation is unlikely to explain our results. Some studies have shown a reduced risk of UL in ever [19], current [27, 30, 47], and longer duration users of oral contraceptives [37, 47]. Most studies found no association between oral contraceptive use and UL risk [26, 28, 29, 31, 39, 48]. Decreased exposure to unopposed estrogen due to the modifying effect of exogenous progestogens has been proposed as a possible explanation [37]; however, the possibility remains that oral contraceptive use may delay the diagnosis of UL by reducing the symptoms, such as heavy menstrual bleeding [33]. Moreover, two studies reported that early initiation of oral contraceptives was associated with an increased risk of UL [27, 28]. In this study, ever use and age at first use of oral contraceptives were not associated with the risk of UL. However, further analyses are warranted to examine the role of the duration and formulation of oral contraceptives in UL development.

This study has several limitations. First, the data on reproductive factors and UL diagnosis were self-reported. Data on reproductive factors were collected before the diagnosis of UL; therefore, misclassification of exposures, including age at menarche and menstrual pattern, were likely non-differential. According to validation studies of self-reported UL in US cohorts, the positive predictive value ranged from 92 to 96% [5, 28, 49]. Although future validation is needed, the medical conditions reported by health professionals are more accurate than those reported by the general population. The cumulative incidence and proportion of UL treatment in our study were comparable to those in previous reports. In the NHIS-NSC, the cumulative incidence of UL over 5 years (2003–2007) was approximately 5%, and the treatment percentage of UL in 2013 was 15% [12]. Before excluding UL cases at baseline, the UL prevalence in our study participants (approximately 10%) was similar to that reported in a previous online survey conducted in 2009 (9%) [13]. Second, detection bias cannot be ruled out. Many UL cases are asymptomatic; however, the cases in this study are likely to be symptomatic because participants in the KNHS were not systemically screened for UL. Incidental detection of UL may be more likely in women with pregnancy, infertility, irregular cycles, and oral contraceptive use than in those without. However, given the inverse association between parity and UL risk, incidental detection is unlikely to explain our results and may attenuate this association. Robust results among women reporting a recent Pap smear screening also suggest that detection bias is unlikely. Third, there is a potential selection bias due to loss to follow-up, even though similar reproductive characteristics were observed between the women who were followed up and those who were not. Fourth, the small number of incident cases limits further analysis, and future confirmation with a larger number of cases is warranted. Fifth, there is a possibility of residual confounding; for example, data on family history of UL and time since last birth were not collected in this study. Finally, the generalizability of our findings to the entire reproductive-age population may be limited, because our population consisted of female nurses aged 22–48 years. However, given the consistency of our results with those of previous studies, there is no strong rationale for differences in the role of reproductive factors in UL development between this study population and women in the general population. Despite these limitations, this is the first longitudinal study to examine the relationship of menstrual cycles and reproductive factors with UL risk in premenopausal Korean women. The availability of information on menstrual patterns at two different points is another strength of our study.

## Conclusions

Findings from this prospective study of female nurses suggest that later age at menarche, long menstrual cycles, long or irregular cycles in early adulthood, and parity are associated with a reduced risk of UL in premenopausal Korean women. The inverse associations of menstruation and parity with UL risk were robust among women reporting a recent gynecological exam. The incidence of UL did not differ according to history of infertility or oral contraceptive use. Our results support the hypothesis that endogenous hormones play a significant role in UL etiology. Further investigation is needed to confirm the long-term effects of menstrual and reproductive factors on UL and other gynecologic conditions.

## Data Availability

The datasets used and analyzed during the current study are available from the corresponding author on reasonable request.

## Abbreviations

UL: Uterine leiomyomata
HR: Hazard ratio
CI: Confidence interval
NHIS-NSC: National Health Insurance Service–National Sample Cohort
KNHS: Korea Nurses’ Health Study
BMI: Body mass index
IQR: Interquartile range
NHS: Nurses’ Health Study
BWHS: Black Women’s Health Study
